# Biolayer Interferometry Analysis for a Higher Throughput Quantification of In-Process Samples of a Rotavirus Vaccine

**DOI:** 10.3390/vaccines10101585

**Published:** 2022-09-21

**Authors:** Sofia B. Carvalho, Mafalda M. Dias, Jean-Philippe Matheise, Isabelle Knott, Patrícia Gomes-Alves, Paula M. Alves

**Affiliations:** 1 iBET, Instituto de Biologia Experimental e Tecnológica, Apartado 12, 2781-901 Oeiras, Portugal; 2 Instituto de Tecnologia Química e Biológica António Xavier, Universidade Nova de Lisboa, Av. da República, 2780-157 Oeiras, Portugal; 3 GSK, Avenue Fleming 20, 1300 Wavre, Belgium; 4 GSK, Rue de l’Institut 89, 1330 Rixensart, Belgium

**Keywords:** rotavirus vaccine, Octet, in-process samples, quantification, bioanalytics

## Abstract

Rotavirus A infection is a global leading cause of severe acute gastroenteritis associated with life-threatening diarrheal episodes in infants and young children. The disease burden is being reduced, namely due to a wider access to rotavirus vaccines. However, there is a demand to expand rotavirus vaccination programs, and to achieve this, it is critical to improve high-throughput in-process product quality control and vaccine manufacturing monitoring. Here, we present the development of an analytical method for the quantification of rotavirus particles contained in a licensed vaccine. The binding of rotavirus proteins to distinct glycoconjugate receptors and monoclonal antibodies was evaluated using biolayer interferometry analysis, applied on an Octet platform. The antibody strategy presented the best results with a linear response range within 2.5 × 10^7^–1.0 × 10^8^ particles·mL^−1^ and limits of detection and quantification of 2.5 × 10^6^ and 7.5 × 10^6^ particles·mL^−1^, respectively. Method suitability for the quantification of in-process samples was shown using samples from different manufacturing stages and their titers were comparable with the approved CC_ID(50)_ method. This cell-free method enables a fast and high-throughput analysis, compatible with time constraints during bioprocess development and it is suitable to be adapted to other viral particle-based drug products.

## 1. Introduction

Rotavirus A infection is a global leading cause of severe acute gastroenteritis associated with life-threatening diarrheal episodes in infants and young children. In 2016, 128,515 deaths were reported worldwide among children younger than 5 years [1]. Moreover, in 2019, 1.76 million hospitalizations were attributed to rotavirus infections [2]. The burden associated with the rotavirus disease is reducing globally due to the implementation of several public health measures and wider access to rotavirus vaccines, with their inclusion in the National Immunization Programs of more than 112 countries worldwide [3]. The FDA approval of the rotavirus vaccines *Rotarix* (a trademark of the GSK group of companies, Rixensart, Belgium) and *RotaTeq* (a trademark of Merck & Co., Pennsylvania, PA, USA) in 2008 has contributed to a significant decrease in the rotavirus prevalence by around 40%. Besides the above vaccines, there are two other prequalified in 2018 [4] by the World Health Organization (WHO) for global use: *Rotasiil* (a trademark of Serum Institute of India, Pune, India) and *Rotavac* (a trademark of.Bharat Biotech, Hyderabad, India).

Vaccination against the rotavirus has been shown to save lives in low-income countries and to be cost-effective or, in some cases, cost-saving in most low- and middle-income countries [5,6]. Different rates of vaccine effectiveness have been reported for different populations and although it remains unclear why this discrepancy is observed, it is most likely due to multiple factors [7,8,9,10,11,12]. Nevertheless, vaccination has a highly positive impact, reducing the burden on public health associated with rotavirus infections. Moreover, the rotavirus vaccine landscape is growing with novel prequalified vaccines and other candidates under development [13]. In this context, new and/or improved analytical tools for high-throughput in-process product quality control (QC) during vaccine development and manufacturing are needed. Rotavirus titer is one of the critical quality attributes (CQA) that needs to be monitored and measured using a robust, specific, and fast analytical method to ensure proper control of rotavirus vaccine production [14]. Potency assays, such as a cell culture infective dose of 50% (CC_ID50_), are the traditional methods accepted by the regulatory authorities for the measurement of infectious particles. These are also currently used for titer quantification. However, these methods present several drawbacks including them being typically time-consuming, labor intensive, and presenting a tendency to be noisy due to the inherent biological variability of cell cultures. The variability can be even higher when the method depends on the subjective observation of cytopathogenic effects (CPE). For rotavirus, CPE is only visible after more than one week [15,16], which is not compatible with in-process analysis during bioprocess development. Other methods, such as PCR or fluorescence-based detection techniques, are more sensitive, allowing early detection (in a few days) and quantification of infected cells [17]. Nevertheless, they are still cell-based assays, also presenting a high variability and lower throughput. During bioprocess development, viral titer quantification of in-process samples in a short-period of time is critical. Therefore, there is a need for analytical tools that allow fast, high-throughput, and robust responses to cope with bioprocess development and manufacturing QC.

Here, we report the development of a high-throughput viral titer quantification method suitable for the analysis of in-process samples of the *Rotarix* rotavirus vaccine. This tool is based on biolayer interferometry (BLI) analyses applied on an Octet platform and takes advantage of the VP7 virus protein binding to a specific monoclonal antibody. BLI surface plasmon resonance techniques can be used as a strategy to quantify viral particles or virus-like particles, offering higher throughput and superior precision when compared to the traditional quantification methods [18,19,20]. The method developed is suitable for the titer analysis of real-time samples from different steps of the purification process, ranging from harvest bulk to purified samples, which are critical for bioprocess monitoring and development. The quantification measurements obtained are in accordance with the results from the standard potency assay method, CCID50. The method developed has the potential to be applied for other rotavirus vaccines, accelerating the vaccine manufacturing pipeline, and contributing to the expansion of vaccination programs worldwide.

## 2. Experimental Section

### 2.1. Samples, Receptors, and Biosensors

Analyzed *Rotarix* vaccine samples were provided by GSK and came from different production batches and purification steps (from harvest bulk, clarification, benzonase treatment, and ultrafiltration).

Glycoconjugated receptors (designed to bind VP8 protein in rotavirus particles) GalNAcα1-3(Fuca1-2)Galb-OCH_2_CH_2_CH_2_NH_2_ (0085-BP) (GlycoNZ, Auckland, New Zealand) and GalNAcα1-3(Fuca1-2)Galb-O(CH_2_)_3_NHCO(CH_2_)_5_NH_2_ (0085a-BP) (GlycoNZ, Auckland, New Zealand), labelled with biotin on a polyacrylic acid backbone, were resuspended to a final concentration of 1 µg·mL^−1^ in Sample Diluent (18-5028, FortéBIO, Pall Corp., Port Washington, NY, USA) according to manufacturer’s instructions. High Precision Streptavidin (SAX) Biosensors (18-0037, FortéBIO, Pall Corp., Port Washington, NY, USA) were used for the glycoconjugated receptor studies. The primary monoclonal antibody α-VP7 (GSK proprietary) was diluted in Sample Diluent and conjugated with Protein A biosensors (18-0004, FortéBIO, Pall Corp., Port Washington, NY, USA).

Recombinant rotavirus VP7 protein (a.a. 51–326) (DAGC101, Creative Diagnostics, Shirley, NY, USA) was used for specific binding assays in the final concentration indicated in the figures.

### 2.2. Sample and Biosensor Preparation

Rotavirus samples were diluted with water (WFI). Ultrafiltered bulk was used as a reference standard with a known concentration (8.0 log10 particles·mL^−1^). Sequential dilutions in WFI were performed to construct the standard curves. Primary antibodies were diluted in Sample Diluent to the final concentrations indicated in the figures and/or in the manuscript body before being loaded into the biosensors. For each biosensor lot, the optimal antibody concentration was assessed. Biosensors were always hydrated and blocked with Sample Diluent for 10 min prior to analysis at room temperature (22 °C).

### 2.3. Biolayer Interferometry Quantification Assay

#### 2.3.1. Biosensors, Receptors, and VP7 Antibodies

Rotavirus binding to glycoconjugated receptors loaded on SAX biosensors was measured by BLI using an OctetRED96 system (FortéBIO, Pall Corp., Port Washington, NY, USA). Rotavirus binding to α-VP7 loaded on Protein A biosensors was measured, as described for SAX biosensors and receptors. Data were acquired in kinetics mode and analyzed using the Data Analysis software v9.0 (FortéBIO, Pall Corp., Port Washington, NY, USA). When necessary, data were exported to a Microsoft Excel file format for further analysis in other software packages.

#### 2.3.2. Quantification Assay Analysis

The quantification assay was set up by diluting the primary antibody or the glycoconjugated receptors with Sample Diluent and loaded into the respective biosensors. Rotavirus samples were then associated with the biosensors and each association profile was measured. The method was defined with five steps: Initial Baseline (120 s), Loading (680 s), Second Baseline (180 s), Association (1200 s), and Dissociation (1200 s). Experiments were performed at 22 °C and sample plates (microplate 96 well, F-bottom, black) (655209, Greiner Bio-One, Kremsmünster, Austria) were continuously agitated at 1000 rpm. All the quantifications were made taking into consideration the response values at equilibrium (1000 to 1200 s) of the binding responses (association step). For data processing, a correction of the association step was performed, removing the first 20 s seconds of the step.

The limit of detection (LOD) and limit of quantification (LOQ) were calculated based on the FDA and EMA Guidelines [21,22]. The approach used for determining both limits was based on the standard deviation of the blank and the slope of the calibration curve:

(1) $$\mathrm{LOD}=\frac{3.3{\;\sigma }_{B}}{S}$$

(2) $$\mathrm{LOQ}=\frac{10{\;\sigma }_{B}}{S}$$

where σ_B_ is the standard deviation of the blank and S is the slope of the calibration curve.

### 2.4. Statistical Analysis

Statistical analysis was performed using GraphPad version 9.1.1 (Dotmatics, San Diego, CA, USA). Differences between the titer obtained with Octet and CC_ID50_ methods were assessed using a Bland−Altman parametric analysis [23]. The Bland−Altman method calculates the differences between both measurements (the mean bias) and the limits of agreement given by the 95% confidence intervals for the mean difference (1.96 × standard deviation). The closest to zero the mean bias is, and the smaller the range between the limits of agreement, the more similar the results evaluated are.

## 3. Results and Discussion

### 3.1. Criteria Definition and Optimization for Assay Implementation

**Figure 1:**
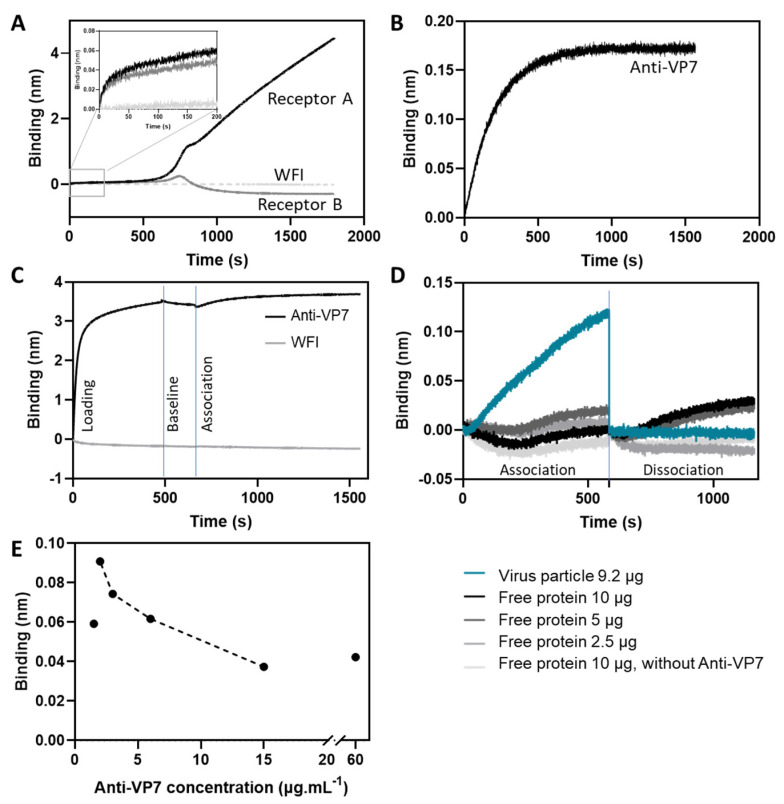
Rotavirus binding response to (**A**) glycoreceptors A and B (receptors’ concentration = 0.5 μg·mL^−1^) and naked streptavidin biosensors (WFI) (representative binding curve, n = 2) and (**B**) anti-VP7 antibody (representative binding curve, n = 3). Primary antibody was diluted to a final concentration of 15 μg ·mL^−1^. Rotavirus sample concentration was 17.6 μg·mL^−1^ (in experiments (**A**–**E**)). (**C**) Evaluation of the specific binding of rotavirus particles to the primary antibody anti-VP7-coated protein A biosensor (black line) in comparison to naked protein A biosensors (grey line). (**D**) Evaluation of free VP7 protein binding. The binding and association profiles of free proteins (3 loads—darker greyscale lines) was compared to the rotavirus sample (9.2 μg—blue line) and with free proteins to the naked biosensor (10 μg—light grey line). Both association and dissociation steps were aligned to 0. (**E**) Antibody titer: different antibody concentrations (ranging from 1.5 to 60 µg·mL^−1^) were evaluated to optimize the binding response (n = 1). Dashed line: concentrations where binding is concentration dependent.

To develop a titer quantification method for in-process samples of a rotavirus vaccine, we used BLI applied on an Octet system. Two approaches were screened: (1) rotavirus’ VP8 protein interaction with two glycoconjugates present in the host cell, 0085-BP (Receptor A) and 0085a-BP (Receptor B) [24] (Figure 1A); and (2) binding of VP7 protein to a specific monoclonal antibody (Figure 1B). To select the best approach, we evaluated the binding magnitude, the association profile, and the data analysis of the processed kinetic data (initial slope versus response at the equilibrium). Rotavirus binding can be measured by both strategies, although different binding response profiles are observed. Both receptors present a low initial binding magnitude, achieving values between 0.06 and 0.05 nm in the first 200 s of the association step for receptors A and B, respectively (Figure 1A). As the binding value tendency was slowly increasing, we extended the association step up to 2000 s. However, although the value increases up to 4 nm for Receptor A, the corresponding binding profile presents a three-step binding transition: the first one from 0 to 580 s, the second from 580 to 820 s, and the last from 820 until the end of the step (1800 s). This is not the standard association curve behavior usually reported for this technique (as observed in Figure 1B for anti-VP7 antibodies) and may suggest receptor conformational changes during the association step and/or biosensor saturation. As for Receptor B, we can also observe a three-step binding transition at lower binding magnitudes when compared to receptor A (achieving a maximum of 0.28 nm), presenting a signal inversion at ≈750 s. Non-specific binding of the rotavirus to the Octet streptavidin biosensors was assessed by measuring the virus association to naked biosensors (without loading of receptors) versus the association observed when the receptors were present. We used WFI to mimic the receptor loading step. As depicted in Figure 1A, no binding occurs to the naked biosensors (WFI), meaning that the blocking step is efficient and that the binding observed (black and dark grey lines) is specific for the receptors used.

The initial binding magnitude of VP7 protein to the monoclonal antibody (from 0 s to 200 s) is higher (0.10 nm) than the one obtained with the receptors (Figure 1B). Moreover, for the time range evaluated (1500 s), it is possible to achieve a stable steady-state response. No multiple transitions were observed. Considering the evaluated parameters, we selected the antibody anti-VP7 strategy to proceed with the assay development. Although the anti-VP7 antibody is highly specific for the rotavirus particles evaluated, we have to consider that for different rotavirus strains, different antibodies may be required. Moreover, further studies using different host receptors, such as integrins and Hsc70 [25], can be performed to evaluate their interaction behavior with the viral proteins as an alternative strategy.

Non-specific binding of the rotavirus to the Octet protein A biosensors was also assessed by measuring the virus association without loading the anti-VP7 antibody versus the association when the antibody was present (Figure 1C). As for Figure 1A, we also used WFI to mimic the loading step. As observed in Figure 1C, no binding occurred to the naked biosensors, meaning that the blocking step was efficient and that the binding observed was specific for the anti-VP7. Additionally, we also evaluated the antibody binding specificity to the whole virus particle compared to a free monomeric recombinant Rotavirus A VP7 protein (Figure 1D). A negligible binding value (≈0.015 nm) was observed for all the loadings of free VP7 protein evaluated (2.5, 5 and 10 μg) using either the anti-VP7 coated biosensor or the naked biosensor (10 μg). Moreover, we can also observe that there is a dissociation of the VP7 free protein samples from the antibody or biosensor characteristic of non-specific binding. Contrarily, the whole rotavirus particle (loading of 9.2 μg, the maximum amount loaded for our undiluted reference standard) presented a higher initial binding rate and binding response for the same time window (0–580 s). No dissociation profile was observed (flat blue line). These results confirm that the binding of the VP7 protein to the selected antibody requires the bioactive form of the virus particles, validating the use of this assay for the evaluation of rotavirus particles’ potency. This observation is in agreement with previous reports of influenza quantification tools using BLI analysis, whereas the native conformational trimeric structure of hemagglutinin protein was found to be critical for receptor interaction [18,26,27,28].

To optimize the binding response obtained, it is critical to define the optimal antibody loading concentration. Several anti-VP7 concentrations were screened using the reference standard to measure the binding responses (Figure 1E). Biosensor saturation for concentrations above 15 μg·mL^−1^ was observed. For concentrations ranging from 2 to 15 μg·mL^−1^, the binding response was dependent on antibody concentration, with the 2 μg·mL^−1^ concentration presenting the optimal (highest) binding value, which is critical to avoid interferences [18,29]. This fine-tuning of the optimal antibody concentration should be considered for each antibody and for each Protein A biosensor lot to avoid lot-to-lot variations.

### 3.2. Assay Performance

**Figure 2:**
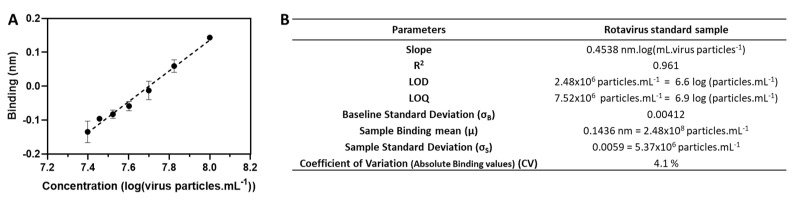
Calibration curve for rotavirus particles binding to anti-VP7 monoclonal antibodies. Error bars represent the standard deviation (representative of n = 3) (**A**). Qualification assay parameters for rotavirus particles based on the response level at equilibrium. Inter-assay precision (for absolute binding values), reported as CV%, was evaluated using a reference standard loaded in three independent runs (**B**).

We used the reference standard (ultrafiltered sample) previously quantified by the limit-dilution CC_ID50_ method to establish a calibration curve. Different virus particle concentrations were obtained through serial dilutions of the sample, ranging from 2.5 × 10^7^ to 1.0 × 10^8^ CC_ID50_·mL^−1^, corresponding to a range between 2.30 and 9.20 µg). Binding responses of the sample dilutions to the anti-VP7 antibody were measured based on the response level at equilibrium (Figure 2A). The assay presented a linear response (R^2^ = 0.961) range from 2.5 × 10^7^ to 1.0 × 10^8^ CC_ID50_·mL^−1^ for the range of concentrations evaluated. The LOD and LOQ calculated are 2.5 × 10^6^ and 7.5 × 10^6^ rotavirus particles·mL^−1^, respectively (Figure 2B). Inter-assay precision was evaluated by analyzing the response of the reference standard that was loaded on three independent runs. The coefficient of variation (CV) obtained is 4.1%, confirming the high precision of the assay. Although the CC_ID50_ CV obtained for the same reference standard is slightly lower (2.5%), the BLI CV is within the CC_ID50_ accepted range for samples from all stages of the bioprocess (from 2.5% up to 5%). The BLI method principle is based on the direct interaction of the virus particles with the antibody. On the other hand, CC_ID50_ takes advantage of the additional virus replication steps, which explains the higher precision obtained. The precision observed for the developed method combined with its real-time analysis capability and higher throughput makes this approach highly promising to cope with the current needs for in-process sample analysis.

### 3.3. Analysis of In-Process Samples

**Figure 3:**
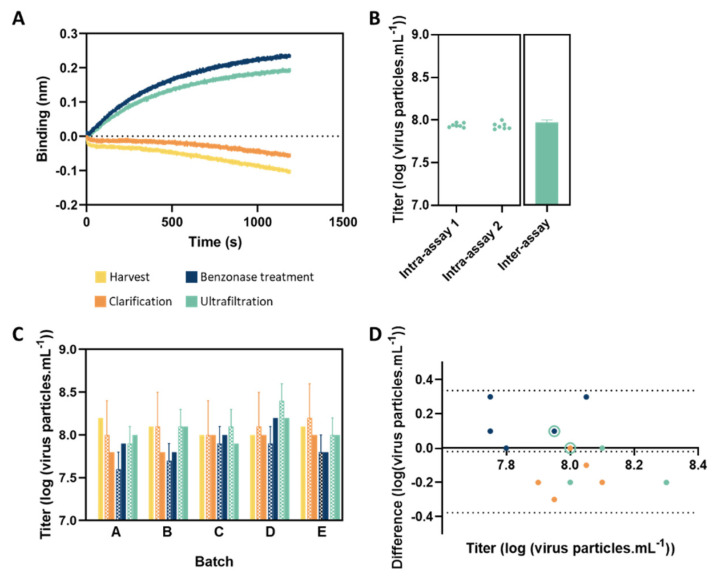
(**A**) Representative binding response of in-process samples from different stages of the bioprocess. (**B**) Method precision and repeatability. Inter-assay precision was evaluated by analyzing three independent sample preparations (ultrafiltered sample) in three independent analyses. Repeatability was assessed in two independent assays analyzing seven independent samples per assay (**C**) In-process sample quantification analysis using CC_ID50_ (pattern bars) or BLI (full bars). Error bars correspond to the standard deviation for the CC_ID50_ method. (**D**) Bland−Altman plot showing the differences between the titer obtained with Octet and CC_ID50_ methods. The dotted lines represent the limits of agreement given by the 95% confidence intervals for the mean bias and the dashed line represents the mean bias from the Bland−Altman plot (the closest to zero, the more similar the results evaluated). Sample Clarified (batch C) (titer 8.0 log, difference 0 log) and sample Benzonase treated (batch C) (titer 7.95 log, difference −0.1 log) overlapped with samples Ultrafiltered (batch E) and Ultrafiltered (batch A), respectively. For visual purposes, the empty circle symbol was used when sample values overlapped. In this case, two ultrafiltrated samples are represented with an open cyan-colored circle.

To evaluate the quantification method, several in-process samples (harvested, clarified, treated with benzonase, and ultrafiltered samples) were analyzed. The same samples (except for harvest samples) were also quantified by CC_ID50_ for titer comparison. Figure 3A shows representative association profiles for the different steps, presenting distinct binding signals and magnitudes, depending on the purification stage. There is a multitude of factors, process, and product-related impurities, that can influence the binding response, such as aggregation, DNA and total protein, or cell culture media components. Here, negative signals of representative binding curves correspond to samples that present lower levels of purity, which impacts the way the light is reflected, changing its interference pattern (Figure 3A,C). These negative signals are common in this technique and the binding signal (magnitude) can be measured as well using either the flip data option (for visualization purposes) or calculating it on the dedicated data analysis software. Harvest and clarified samples are also more suitable to present higher errors associated with the titration for both methods. To determine the assay precision for titer quantification, we investigated the inter- (assay-to-assay) and intra-assay (repeatability) variability (Figure 3B). Inter-assay precision (CV = 0.37%) was evaluated by analyzing three independent sample preparations (ultrafiltered sample) in three independent analyses. Repeatability was assessed in two independent assays where we analyzed seven independent samples per assay (CV_Intra-assay1_ = 0.29% and CV_Intra-assay2_ = 0.30%). The CVs obtained are lower than 0.4%, demonstrating a high precision and acceptable variability of the method. As we can observe in Figure 3C, this BLI method allows the quantification of samples from distinct steps of the purification process, as well as batch-to-batch comparisons. The titer values obtained for both methods presented similar values, which were confirmed by the Bland−Altman plot (Figure 3D). The mean bias obtained is very small (−0.02) and all the measurements fall between the limits of agreement (from −0.38 to 0.34 log). Therefore, the quantification variations obtained between the two methods are within the acceptable range (±0.4 log) previously defined for the CC_ID50_ analysis of these in-process samples. For the standard reference (purified sample (ultrafiltered)), the differences observed are between 0 and −0.2 log, which is also in agreement with the CC_ID50_ thresholds (±0.2 log) generally defined at method validation for CC_ID50_ tests. Harvest samples were not evaluated by CC_ID50_ and, therefore, cannot be compared by the Bland−Altman plot. We can also observe that the difference signal is always positive (benzonase treatment) or negative (clarification and ultrafiltration), depending on the sample group. This suggests that titers for clarified and ultrafiltered samples tend to be higher when measured by the Octet method and that benzonase treated sample titers tend to be lower when compared to the CC_ID50_. The robustness of this comparison can be improved by measuring more replicates. Implementation of different calibration curves using standards for specific steps of the bioprocess can be helpful to improve even further the BLI CV and the comparability between methods.

## 4. Conclusions

Cell-based methods are still considered the gold-standard for viral particle titration. However, to cope with the demanding need for improved bioanalytics for high-throughput product quality control and vaccine manufacturing, we developed a BLI assay for the monitoring and quantification of rotavirus vaccine bioprocess samples. This method presents a wide linear dynamic range and a LOQ suitable for the quantification of in-process samples. Assay specificity and precision performance are comparable to the gold standard CC_ID50_ method. Titration using BLI enables faster results (hours vs. days) and higher throughput while requiring fewer human resources and associated costs, thus being compatible with the time constraints during bioprocess development. Vaccine analytics face increasing demands as the vaccine landscape is constantly evolving. Faster, higher-throughput, and automated methods are needed to support in-process product quality control for vaccine development and manufacturing. These methods can streamline current vaccine production processes or the development of new vaccines by shortening the testing times. Moreover, they can also be critical to solve manufacturing issues that can delay vaccine production, impacting their supply. Besides its potential to improve rotavirus process characterization and excelling manufacturing pipelines of currently marketed vaccines, it can also be adapted to other serotypes, other types of rotavirus vaccines, or other viral particle-based drug products.

## Data Availability

The data presented in this study are available from the corresponding author upon reasonable request. Some of the data cannot be publicly available due to confidentiality issues.

## Abbreviations

BLI, biolayer interferometry; CC_ID50_, cell culture infective dose 50%; CPE, cytopathogenic effects; CQA, critical quality attributes; EMA, European Medicines Agency; FDA, Food and Drug Administration; LOD, limit of detection; LOQ, limit of quantification; QC, quality control; SAX, high precision streptavidin; WHO, World Health Organization; WFI, water for injection.

## References

[1] Troeger C., Khalil I.A., Rao P.C., Cao S., Blacker B.F., Ahmed T., Armah G., Bines J.E., Brewer T.G., Colombara D.V. (2018). Rotavirus Vaccination and the Global Burden of Rotavirus Diarrhea Among Children Younger Than 5 Years. JAMA Pediatr..

[2] Hallowell B.D., Chavers T., Parashar U., Tate J.E. (2022). Global Estimates of Rotavirus Hospitalizations Among Children Below 5 Years in 2019 and Current and Projected Impacts of Rotavirus Vaccination. J. Pediatric. Infect Dis. Soc..

[3] World Health Organization Vaccine in National Immunization Programme Update. Www.Who.Int/Immunization/Monitoring_surveillance/VaccineIntroStatus.Pptx?Ua=1.

[4] Burke R.M., Tate J.E., Kirkwood C.D., Steele A.D., Parashar U.D. (2019). Current and New Rotavirus Vaccines. Curr. Opin. Infect. Dis..

[5] Buttery J.P., Kirkwood C. (2021). Rotavirus Vaccine Implementation: Evidence to Fill the Gap?. Lancet Glob. Health.

[6] Newall A.T., Leong R.N., Reyes J.F., Curns A.T., Rudd J., Tate J., Macartney K., Parashar U. (2021). Rotavirus Vaccination Likely to Be Cost Saving to Society in the United States. Clin. Infect. Dis..

[7] Burke R.M., Tate J.E., Parashar U.D. (2021). Global Experience With Rotavirus Vaccines. J. Infect Dis..

[8] Karafillakis E., Hassounah S., Atchison C. (2015). Effectiveness and Impact of Rotavirus Vaccines in Europe, 2006–2014. Vaccine.

[9] Kirkwood C.D., Ma L.-F., Carey M.E., Steele A.D. (2019). The Rotavirus Vaccine Development Pipeline. Vaccine.

[10] Kollaritsch H., Kundi M., Giaquinto C., Paulke-Korinek M. (2015). Rotavirus Vaccines: A Story of Success. Clin. Microbiol. Infect..

[11] Snelling T.L., Andrews R.M., Kirkwood C.D., Culvenor S., Carapetis J.R. (2011). Case-Control Evaluation of the Effectiveness of the G1P [8] Human Rotavirus Vaccine during an Outbreak of Rotavirus G2P [4] Infection in Central Australia. Clin. Infect. Dis..

[12] Tate J.E., Ngabo F., Donnen P., Gatera M., Uwimana J., Rugambwa C., Mwenda J.M., Parashar U.D. (2016). Effectiveness of Pentavalent Rotavirus Vaccine Under Conditions of Routine Use in Rwanda. Clin. Infect. Dis..

[13] Wang Y., Li J., Liu P., Zhu F. (2021). The Performance of Licensed Rotavirus Vaccines and the Development of a New Generation of Rotavirus Vaccines: A Review. Hum. Vaccines Immunother..

[14] Sanyal G., Särnefält A., Kumar A. (2021). Considerations for Bioanalytical Characterization and Batch Release of COVID-19 Vaccines. NPJ Vaccines.

[15] Le T.T., Cramer J.P., Chen R., Mayhew S. (2020). Evolution of the COVID-19 Vaccine Development Landscape. Nat. Rev. Drug Discov..

[16] Thanh Le T., Andreadakis Z., Kumar A., Gómez Román R., Tollefsen S., Saville M., Mayhew S. (2020). The COVID-19 Vaccine Development Landscape. Nat. Rev. Drug Discov..

[17] Ranheim T., Mathis P.K., Joelsson D.B., Smith M.E., Campbell K.M., Lucas G., Barmat S., Melissen E., Benz R., Lewis J.A. (2006). Development and Application of a Quantitative RT-PCR Potency Assay for a Pentavalent Rotavirus Vaccine (RotaTeq^®^). J. Virol. Methods.

[18] Carvalho S.B., Moleirinho M.G., Wheatley D., Welsh J., Gantier R., Alves P.M., Peixoto C., Carrondo M.J.T. (2017). Universal Label-Free in-Process Quantification of Influenza Virus-like Particles. Biotechnol. J..

[19] Durous L., Julien T., Padey B., Traversier A., Rosa-Calatrava M., Blum L.J., Marquette C.A., Petiot E. (2019). SPRi-Based Hemagglutinin Quantitative Assay for Influenza Vaccine Production Monitoring. Vaccine.

[20] Nilsson C.E., Abbas S., Bennemo M., Larsson A., Hämäläinen M.D., Frostell-Karlsson Å. (2010). A Novel Assay for Influenza Virus Quantification Using Surface Plasmon Resonance. Vaccine.

[21] European Medicines Agency (1995). ICH Topic Q 2 (R1) Validation of Analytical Procedures: Text and Methodology (CPMP/ICH/381/95).

[22] Food and Drug Administration (1996). ICH Guidance for Industry, Q2B Validation of Analytical Procedures: Methodology (FDA-1996-D-0169).

[23] Bland J., Altman D. (1986). Statistical Methods for Assessing Agreement between Two Methods of Clinical Measurement. Lancet.

[24] Stencel-Baerenwald J.E., Reiss K., Reiter D.M., Stehle T., Dermody T.S. (2014). The Sweet Spot: Defining Virus–Sialic Acid Interactions. Nat. Rev. Microbiol..

[25] Desselberger U. (2014). Rotaviruses. Virus Res..

[26] Bodle J., Verity E.E., Ong C., Vandenberg K., Shaw R., Barr I.G., Rockman S. (2013). Development of an Enzyme-Linked Immunoassay for the Quantitation of Influenza Haemagglutinin: An Alternative Method to Single Radial Immunodiffusion. Influenza Other Respir. Viruses.

[27] Khurana S., King L.R., Manischewitz J., Coyle E.M., Golding H. (2014). Novel Antibody-Independent Receptor-Binding SPR-Based Assay for Rapid Measurement of Influenza Vaccine Potency. Vaccine.

[28] Vachieri S.G., Xiong X., Collins P.J., Walker P.A., Martin S.R., Haire L.F., Zhang Y., McCauley J.W., Gamblin S.J., Skehel J.J. (2014). Receptor Binding by H10 Influenza Viruses. Nature.

[29] Pall Life Sciences Biomolecular Binding Kinetics Assays on the Octet Platform Application Note 14. http://separations.co.za/wp-content/uploads/2017/05/OCTET.pdf.

